# Is sleep quality a moderated mediator between perceived stress and depression among stroke patients?

**DOI:** 10.3389/fpubh.2023.1284197

**Published:** 2024-01-05

**Authors:** Lina Guo, Miao Wei, Genoosha Namassevayam, Mengyv Zhang, Yvying Xie, Runtang Meng, Yuanli Guo, Yanjin Liu

**Affiliations:** ^1^Department of Neurology, National Advanced Stroke Center, The First Affiliated Hospital of Zhengzhou University, Zhengzhou, China; ^2^Department of Supplementary Health Sciences, Faculty of Health-Care Sciences, Eastern University, Chenkalady, Sri Lanka; ^3^School of Nursing and Health, Zhengzhou University, Zhengzhou, China; ^4^School of Public Health, Hangzhou Normal University, Hangzhou, China; ^5^Department of Nursing, The First Affiliated Hospital of Zhengzhou University, Zhengzhou, China

**Keywords:** stroke, sleep quality, perceived stress, depression, social support, mediating effect, moderating effect

## Abstract

**Background:**

Sleep quality can offer new insights into addressing depression among stroke patients. However, the current understanding of the mechanism by which sleep quality reduces depression is not clear in existing research.

**Objectives:**

This study aimed to identify the relationships and mechanisms among perceived stress, sleep quality, social support, and depression in stroke patients.

**Methods:**

A multicenter cross-sectional study was conducted from January to May 2023. Cluster random sampling was used to recruit 500 stroke patients from five hospitals in Henan Province, China. The Chinese Perceived Stress Scale (CPSS), Pittsburgh Sleep Quality Index (PSQI), Social Support Rating Scale (SSRS), and Hamilton Depression Scale (HAMD-24) were employed to assess perceived stress, sleep quality, social support, and depression, respectively. Data were analyzed using descriptive analysis, *Pearson’s* correlation analysis, and moderated mediation analysis. The study adhered to the STROBE checklist for reporting.

**Results:**

Out of 500 participants, 471 completed the survey (94.2%). After controlling for sex and age, mediation analysis revealed that poor sleep quality partially mediated the relationship between perceived stress and depression (*β* = 0.184, 95% CI: 0.110, 0.359). Additionally, social support played a moderating role in the mediation model.

**Conclusion:**

This study explained the moderated mediation of sleep quality and social support between perceived stress and depression. It provided a theoretical basis for the development of a sleep quality intervention program for reducing depression among stroke patients.

## Introduction

Stroke is the second-leading cause of death worldwide, with its prevalence, morbidity, and overall impact steadily increasing each year (1). The estimated worldwide prevalence of stroke is 1,240 per 100,000 individuals (2). According to the Global Burden of Disease study (GBD), there were 101 million reported cases of stroke worldwide in 2019 (3, 4). Furthermore, stroke accounted for 143 million disability-adjusted life years (DALYs), constituting 5.7% of the total burden of diseases (5, 6). The incidence of stroke cases in China has consistently risen over time (7). Numerous studies have indicated that over 70% of individuals who have experienced a stroke continue to manifest significant levels of impairment or disability. This results in limited self-care function and increased susceptibility to acute disorders, followed by a range of psychological problems. Among these, perceived stress and depression are the most prevalent.

Perceived stress refers to the extent of stress an individual experiences from an external event that surpasses self-absorption (8). Population-based epidemiological studies have indicated that stroke patients are prone to experiencing perceived stress, and it may serve as a reliable predictor of depressive symptoms (9–12). Depression is a common complication following a stroke attack (13). Post-stroke depression (PSD) refers to a syndrome that emerges after a stroke event, encompassing a range of depressive symptoms and associated somatic manifestations (14). Approximately one-third of stroke patients develop depression at varying intervals following an early stroke, with a cumulative incidence ranging from 39 to 52% over 5 years. In the United States, 20–65% of stroke patients experience depression (15). Depression significantly impairs their ability to perform daily activities and exacerbates cognitive dysfunction in stroke patients (16, 17). The mortality rate among stroke patients with depression is 3.4 times higher than that of those without depression, a phenomenon known as the double burden of stroke (18).

It is noteworthy that sleep quality can play a pivotal role in reducing perceived stress, depression, and overall recovery following a stroke event (19, 20). Nevertheless, research indicates a high prevalence of poor sleep quality in stroke patients, reaching up to 30.1% (21). Furthermore, persistent poor sleep quality has detrimental effects on neurological function recovery, overall quality of life, and physical and mental wellbeing. Most concerning is its association with an increased risk of mortality and stroke recurrence. Empirical evidence has established poor sleep quality as a distinct risk factor for depression, with more than 90% of individuals with depression experiencing sleep problems (22). Individuals who achieve good sleep quality exhibit a 10–30% lower risk of cardiovascular disease than those with poor sleep quality (23). Results from a randomized controlled experiment indicated that intervening in sleep quality could reduce 87% of depressive symptoms (24). Despite this, there is a notable lack of emphasis on the importance of sleep quality among stroke patients.

Moreover, the provision of social support has been shown to positively impact perceived stress and depression. Social support, encompassing understanding, recognition, and assistance from familial, friendly, and other communal networks, plays a crucial role in individuals’ wellbeing (25). There exists a significant positive relationship between perceived stress and depression, and effective social support has the potential to alleviate these conditions among stroke patients (26). Moreover, social support may contribute to the improvement of sleep quality by reducing perceived stress and, consequently, lowering the incidence of depression among stroke patients.

American psychologist Lazarus’ stress and coping theory points out that stress is a product of the interaction between humans and the environment (27). When internal and external environmental stimuli exceed one’s own coping ability and resources, stress will be generated. If the intensity of the stressors is high or persistent, and the stress cannot be successfully relieved, corresponding emotional or behavioral reactions will occur. The confirmed relationship between sleep quality and depression among students, with sleep quality acting as a mediator and social support as a moderator, has been documented (28). However, there is limited knowledge regarding these dynamics among stroke patients. The established theory not only provides a robust foundation for elucidating the antecedents of depression but also furnishes a comprehensive theoretical framework. The deleterious impact of stress on depression may be mediated and moderated through various mechanisms. One such avenue for explaining these mechanisms is the interplay between sleep quality and social support. Consequently, this study aimed to delve into these mechanisms based on the observed correlation, enlightening the relationships between sleep quality, social support, and depression.

**Figure 1:**
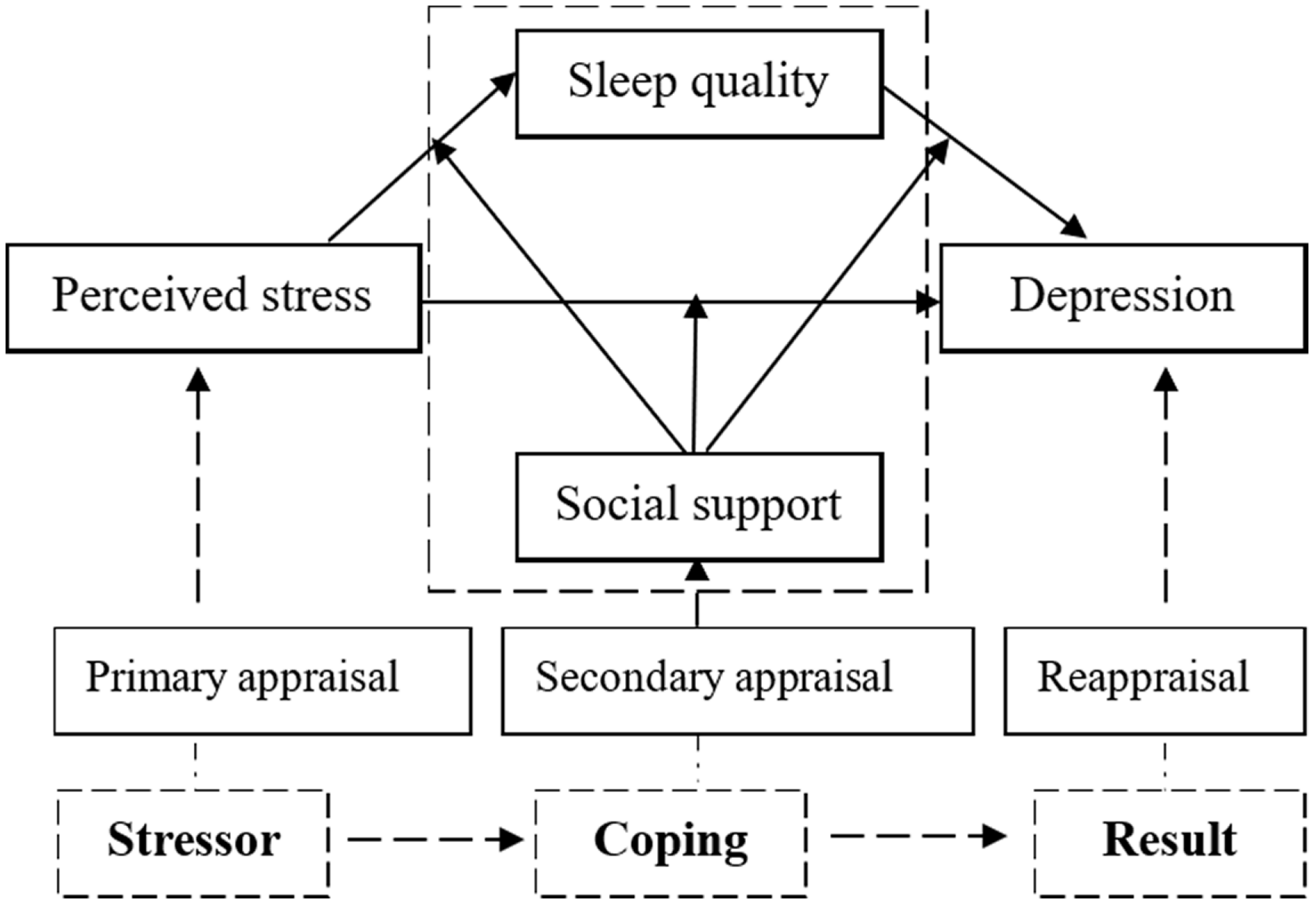
Hypothesis theoretical model of moderated mediation.

Based on this theory, the following research hypotheses are proposed: Hypothesis 1, there is a relationship between perceived stress, sleep quality, depression, and social support. Hypothesis 2, sleep quality has a mediating effect between perceived stress and depression; Hypothesis 3, social support has a moderating effect between perceived stress, sleep quality, and depression. The theoretical framework is presented in Figure 1.

Therefore, the purpose of this study was to explore the moderated mediating mechanism of sleep quality and social support among stroke patients and to provide theoretical guidance for subsequent intervention studies. This will aid in understanding psychological phenomena related to sleep and guide healthcare professionals in improving sleep quality in post-stroke patients.

## Methods

### Participants and procedure

Patients were eligible for inclusion in the study if they met the following criteria: (1) had a physician-diagnosed stroke; (2) were at least 18 years old; (3) demonstrated effective communication skills; and (4) exhibited normal cognitive function, with a score of 24 or higher on the Mini-Mental State Examination (29). Individuals were excluded from the study if they: (1) had previously participated in other research studies; or (2) suffered from severe organ dysfunctions or physical illnesses not suitable for investigation.

A total of 500 participants were selected for this study from five tertiary grade A hospital in Henan Province, China. Following recommended guidelines for cross-sectional surveys, the sample size ideally should be 10–20 times the total number of variables, with a provision for a 20% allowance for invalid questionnaires. Given the inclusion of 24 independent variables in the study, the calculated sample size ranged from 300 to 600 participants. Ultimately, the survey was completed by 471 stroke survivors, resulting in an effective response rate of 94.2%.

Moreover, it is crucial to emphasize that this study strictly adhered to the STROBE (Strengthening the Reporting of Observational Studies in Epidemiology) statement checklist. This checklist is designed to establish essential criteria that enhance the transparency and quality of reporting in observational studies, ensuring the completeness and reliability of the research findings.

### Ethical considerations

Ethical approval for this study was obtained from the Ethics Committee of the First Affiliated Hospital of Zhengzhou University (2022-KY-1168-001). Prior to participation, each participant provided written informed consent, signifying their voluntary involvement and understanding of the study’s procedures, potential risks, benefits, and the confidentiality of their data.

### Data collection

The cluster-random sampling approach was utilized to collect data from January 2023 to May 2023. Henan Province was initially divided into five distinct regions: eastern, southern, western, northern, and central. Within each region, tertiary grade A hospitals were numbered. To ensure randomness, we employed an online random number generator^1^ to select one hospital from each region for participation in the survey. Consequently, five tertiary grade A hospitals were recruited for the study.

Approval and consent were obtained from the respective hospital managers. To ensure standardized data collection procedures, nurses involved in the survey received comprehensive training. This training covered the use of each measurement scale and the application of consistent instructions and language during the investigations. Before conducting the survey, nurses provided detailed explanations to the patients, outlining the purpose, significance, and methodology of completing the questionnaire. Throughout the survey process, investigators promptly addressed any concerns or queries raised by the patients. In instances where questionnaires had missing or empty items, they were completed on the spot. For cases in which patients faced difficulty completing the questionnaire independently, whether due to factors such as educational level or visual impairment, the investigator filled in the questionnaire based on the patient’s responses. After the survey, the collected questionnaires were carefully examined on-site to enhance data accuracy and completeness. This quality control process aimed to ensure the reliability of the collected information.

### Measurements

The researchers designed a social-demographic information questionnaire, which included inquiries about age, sex, body mass index (BMI), education, work status, residence, residential status, medical insurance type, marital status, monthly income, number of children, and history of smoking and alcohol consumption. Disease-related characteristics, such as the history of stroke, TOAST classification, times of stroke attacks, thrombolysis status, duration after the first stroke, and the Charlson Comorbidity Index (CCI), were extracted from the medical records. Additionally, stroke severity information was obtained from the same records using two widely recognized assessment scales: The National Institute of Health Stroke Scale (NIHSS) and the modified Rankin Scale (mRS).

The evaluation of perceived stress symptoms was conducted using the Chinese version of the Perceived Stress Scale (CPSS), originally developed by Cohen (30). This scale comprises 14 items organized into two dimensions: a sense of being out of control and a feeling of tension. All items were rated on a 5-point Likert scale ranging from 0 to 4, with a maximum score of 56. A higher score indicated a higher level of perceived stress (31).

Sleep quality over the last month was assessed using the widely employed Pittsburgh Sleep Quality Index (PSQI) (32), known for its good reliability and validity (33). The 18-item PSQI includes seven dimensions: sleep quality, sleep disturbance, habitual sleep efficiency, sleep latency, sleep disturbances, hypnotic medication use, and daytime dysfunction. Each item had a score range from 0 to 3, with a higher score indicating poorer sleep quality. Cronbach’s alpha for reliability in this study was 0.863.

Social support was evaluated using the Social Support Rating Scale (SSRS), developed by Chinese researcher Xiao (34). This scale, comprising 10 items and three dimensions (subjective support, objective support, and utilization of social support), had a total possible score ranging from 12 to 66. A higher score indicated greater social support (35), with a threshold of 23 or less considered as low social support. Cronbach’s alpha for reliability in this study was 0.819.

The severity of depressive symptoms was assessed using the 24-item Hamilton Depression Scale (HAMD-24) (36). The HAMD-24 comprises seven dimensions: anxiety/somatization, weight, cognitive disturbance, diurnal variation, retardation, sleep disturbance, and hopelessness. Most items use a 5-point scale ranging from 0 to 4, with a few items using a 3-point scale ranging from 0 to 2. The total score ranges from 0 to 76, with a higher score indicating more severe depression (37). Cronbach’s alpha for reliability in this study was 0.823.

The statistical analysis was conducted using SPSS 21.0 (IBM Corporation, Armonk, NY, USA). Continuous variables were presented as means and standard deviations, while categorical variables were described with frequencies and percentages. The Pearson correlation coefficient was used to assess the relationships between stress, depression, sleep quality, and social support. Hierarchical regression analysis, specifically Model 59 of the SPSS-PROCESS procedure (38, 39) was employed to investigate mediation, moderation, and moderated mediation among stress, sleep quality, social support, and depression. Age and sex were considered as covariates, and the analysis used 5,000 bootstrap samples for replications. A significance level of *p* < 0.05 was applied to determine statistical significance.

## Results

### Descriptive characteristics

The age of the 471 stroke survivors in this study ranged from 23 to 88 years, with a mean age of 58.28 years (SD = 15.49). The BMI had a mean value of 24.56 (SD = 2.50). The mean scores for the SSRS, CPSS, and HAMD-24 were 39.11 (SD = 6.53), 24.46 (SD = 6.99), and 12.61 (SD = 3.41), respectively. Importantly, the mean value of the PSQI was 7.42 (SD = 4.22), indicating that 70.90% of the patients experienced sleep problems. Additional characteristics of the participants are presented in Table 1.

**Table 1:** Characteristics of the sample (*n* = 471).

Variables	*n* (%)	Variables	*n* (%)
Sex		Smoking	
Male	328 (69.6)	Non-smoker	254 (53.9)
Female	143 (30.4)	Current smoker	146 (31.0)
Education		Former smoker	71 (15.1)
Elementary school or below	193 (41.0)	Drinking	
Junior school	137 (29.1)	Non-drinker	275 (58.4)
High school	81 (17.2)	Current drinker	142 (30.1)
Undergraduate and above	60 (12.7)	Former drinker	54 (11.5)
Monthly income (RMB)		Employment status	
<3,000	265 (56.3)	Unemployed	148 (31.4)
3,000–5,000	140 (29.7)	Employed	227 (48.2)
>5,000	66 (14.0)	Retired	96 (20.4)
Spouse		Duration after first stroke	
Have	390 (82.8)	≤ 3 months	198 (42.0)
No	81 (17.2)	≤ 1 year	149 (31.6)
Residence		≤ 3 years	56 (11.9)
Rural	335 (71.1)	>3 years	68 (14.4)
Urban	136 (28.9)	mRs	
Residential status		≤2	286 (60.7)
Live alone	27 (5.7)	>2	185 (39.3)
Live with spouse	200 (42.5)	CCI	
Live with children	37 (7.9)	≤2	268 (56.9)
Live with spouse and children	207 (43.9)	>2	203 (43.1)
Health insurance type		Thrombolysis	
Urban residents basic health insurance	102 (21.7)	Yes	413 (87.7)
Rural residents basic health insurance	198 (42.0)	No	58 (12.3)
Employee basic health insurance	46 (9.8)	TOAST	
Retired cadres health insurance	73 (15.5)	Large-artery atherosclerosis	204 (43.3)
Commercial insurance	32 (6.8)	Cardioembolism	38 (8.1)
Other	20 (4.2)	Small-artery occlusion	108 (22.9)
Number of children		Other/unknown	121 (25.7)
0	45 (9.6)	Occurrence of stroke attack	
1	58 (12.3)	First time	277 (58.8)
2	236 (50.1)	Second times	144 (30.6)
≥3	132 (28.0)	≥3 time	50 (10.6)
Family history of stroke		NIHSS	
Yes	126 (26.8)	≤4	301 (63.9)
No	345 (73.2)	>4	170 (36.1)

### Correlation analysis

*Pearson* correlation analyses revealed that perceived stress was positively correlated with poor sleep quality (*r* = 0.572, *p* < 0.01) and depressive symptoms (*r* = 0.648, *p* < 0.01). Poor sleep quality also showed a positive association with depression (*r* = 0.486, *p* < 0.01). Conversely, social support exhibited a significantly negative correlation with perceived stress (*r* = −0.421, *p* < 0.01), poor sleep quality (*r* = −0.328, *p* < 0.01), and depression (*r* = −0.354, *p* < 0.01). To address the issue of multiple testing, *R* (version 4.2.0; 40) was used for false discovery rate (FDR) correction (Table 2).

**Table 2:** Correlation of main variables (*n* = 471).

Variables	Perceived stress	Poor sleep quality	Social support	Depression
Perceived stress	1			
Poor sleep quality	0.572^**#^	1		
Social support	−0.0421^**#^	−0.0328^**#^	1	
Depression	0.648^**#^	0.486^**#^	−0.354^**#^	1

**p < 0.01; ^#^FDR (false discovery rate) < 0.01.

### Mediation analysis

As shown in Tables 3 4, with sex and age as controlled variables, the results of the mediation analysis revealed that perceived stress positively and indirectly predicted depression through poor sleep quality (*β* = 0.184, *p* < 0.001). The 95% bias-corrected bootstrap confidence interval was 0.110–0.359, signifying that the indirect effect of perceived stress on depressive symptoms was statistically significant. Furthermore, the direct effect of perceived stress on depressive symptoms (*β* = 0.338, *p* < 0.001) was also significant, indicating that poor sleep quality partially mediated the relationship between perceived stress and depression.

**Table 3:** Results of poor sleep quality as a mediator (*n* = 471).

Model	Outcome variable	Independent variables	*β*	*p*
Model 1	Poor sleep quality	Constant	−4.671	0.000
		Perceived stress	0.482	0.000
		Sex	0.238	0.426
		Age	0.003	0.773
		*R^2^*	0.659***	
		*F*	125.974	
Model 2	Depression	Constant	−8.44	0.000
		Perceived stress	0.522	0.000
		Sex	0.078	0.879
		Age	−0.020	0.333
		*R^2^*	0.436***	
		*F*	50.511	
Model 3	Depression	Constant	−6.659	0.001
		Perceived stress	0.338	0.000
		Poor sleep quality	0.381	0.002
		Sex	−0.013	0.979
		Age	−0.021	0.291
		*R^2^*	0.436***	
		*F*	50.511	

***p < 0.001.

**Table 4:** The total, direct, and indirect effects of the mediation model (*n* = 471).

	*β*	*SE*	*t*	*p*	LL95%CI	UL95%CI
Total effect	0.522	0.043	12.251	0.001	0.438	0.606
Direct effect	0.338	0.071	4.75	0.001	0.198	0.479
Indirect effect	0.184	0.063	/	/	0.110	0.359

LL95%CI, low limit of 95% confidence interval; UL95%CI, under limit of 95% confidence interval.

### Moderated mediation analysis

**Figure 2:**
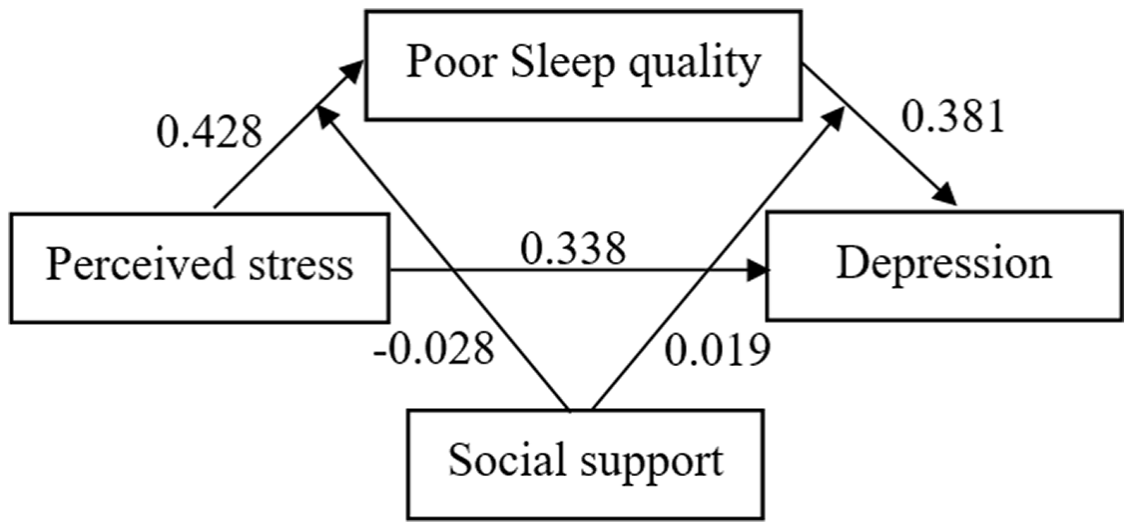
Moderated mediation model.

The moderating effect of social support indicated that the interaction between perceived stress and social support contributed to poor sleep quality (*β* = −0.028, 95% CI: −0.054, −0.003), and the interaction between poor sleep quality and social support contributed to depression (*β* = 0.019, 95% CI: 0.004, 0.034). However, the results of the moderated mediation analysis revealed that social support did not play a moderating role in the direct effect between perceived stress and depression (*β* = −0.018, 95% CI: −0.046, 0.011). Detailed information is given in Table 5, and the final moderated mediation model is displayed in Figure 2.

**Table 5:** The moderated mediation analysis (*n* = 471).

Outcome variable	Independent variables	*β*	*p*	LL95%CI	UL95%CI
Poor sleep quality	Constant	−0.360	0.814	−3.378	2.657
	Perceived stress	1.017	0.000	0.054	0.782
	Social support	−0.018	0.000	−0.131	0.095
	Interaction 1	−0.028	0.030	−0.054	−0.003
	Age	0.019	0.423	−0.028	0.066
	Sex	−0.798	0.187	−1.986	0.390
	*R^2^*	0.518 ***			
	*F*	41.766			
Depression	Constant	6.974	0.000	4.686	9.262
	Perceived stress	0.471	0.000	0.262	0.680
	Poor sleep quality	0.214	0.000	0.106	0.321
	Social support	−0.201	0.000	−0.287	−0.115
	Interaction 1	−0.018	0.223	−0.046	0.011
	Interaction 2	0.019	0.012	0.004	0.034
	Age	−0.008	0.652	−0.04	0.028
	Sex	0.027	0.953	−0.880	0.934
	*R^2^*	0.568***			
	*F*	36.078			

***p < 0.001; Interaction 1, perceived stress× social support; Interaction 2, poor sleep quality ×social support; LL95%CI, low limit of 95% confidence interval; UL95%CI, under limit of 95% confidence interval.

To further validate the moderating effect of social support, we categorized it into three groups: low (mean minus one SD), moderate (mean), and high (mean plus one SD). As presented in Table 6, poor sleep quality significantly mediated the association between perceived stress and depression at both moderate (*β* = 0.217, 95% CI: 0.032, 0.459) and high levels of social support (*β* = 0.280, 95% CI: 0.038, 0.788). Tables 5 6, along with Figure 2, illustrate that at moderate and higher levels of social support, an increase in social support was associated with a weaker relationship between perceived stress and sleep, while the connection between poor sleep quality and depression became stronger.

**Table 6:** Conditional indirect effects of perceived stress on depression at different values of social support (*n* = 471).

Path	Moderator (Social support)	*β*	SE	LL95%CI	UL95%CI
Perceived stress→ Poor sleep quality → Depression	Low	0.111	0.132	−0.097	0.434
	Medium	0.217	0.106	0.032	0.459
	High	0.280	0.193	0.038	0.788

## Discussion

This study represents the first exploration of the relationship between perceived stress, sleep quality, social support, and depression using mediating and moderating effects analyses among stroke patients in a multicenter survey in China. The application of the moderated mediation analysis method offers valuable methodological guidance for future nursing research. The identification of relationships and mechanisms among these four variables provides a theoretical framework for future clinical nursing interventions.

The correlations observed among perceived stress, sleep quality, social support, and depression in stroke patients support Hypothesis 1. Specifically, a higher presence of perceived stress symptoms was associated with an increased risk of depression, while better sleep quality and stronger social support were linked to a reduced risk of depressive symptoms. This finding aligns with a previous study conducted among older stroke patients (41), which similarly found a significant and positive correlation between perceived stress and depression. Additionally, a longitudinal study based on 18,776 participants, followed for 11.8 years from the UK Biobank database, revealed that good sleep quality was significantly associated with a 55% lower risk of anxiety and depression (42). Coincidentally, a research team conducting both quantitative and qualitative studies highlighted the crucial role of social support in overcoming anxiety and depression (43, 44). However, it is interesting to note that a study on stroke patients also affirmed the correlation between sleep quality, stress, depression, and social participation. Yet, the path analysis in that article indicated that depression serves as a risk factor for sleep quality, presenting a different conclusion from our study (45). This suggests the presence of a complex, potentially bidirectional interaction between sleep quality and stress.

The results from PROCESS analysis validated the theoretical hypothesis regarding the moderated mediating mechanism of sleep quality and social support among stroke patients. Sleep quality acted as a mediating variable between stress and depression, with poor sleep quality exacerbating the impact of perceived stress on depression and better sleep quality mitigating the impact of stress on depression. This finding aligns with Hypothesis 2, suggesting that when individuals experience stress, the generation of pressure depends on cognitive evaluation and coping, and the quality of sleep reflects the effectiveness and appropriateness of these processes. This is consistent with a study among older people by Liu and colleagues (46), which also supported this conclusion. At moderate and high levels of social support, it has been confirmed that social support moderates the relationship between perceived stress and sleep quality, as well as between poor sleep quality and depression. As expected, higher social support weakened the connection between perceived stress and poor sleep quality among stroke patients. This may be because patients with greater social support are less likely to experience perceived stress and depression in response to the same stressors. This protective role of social support has also been observed in breast cancer patients, where social support played a protective role in anxiety, depression, and sleep quality (47). Most notably, even at higher levels of social support, stroke patients with poor sleep quality were found to be more sensitive to experiencing depressive symptoms. This is likely due to the fact that poor sleep quality can lead to physiological disorders and emotional anxiety, further aggravating depressive symptoms. This result is in line with previous research (48, 49) and provides additional support for the validity of Hypothesis 3.

Therefore, this study offers the following insights: First, attention should be directed toward alleviating the perceived stress of stroke patients. This can be achieved by reducing stressors to lower their perceived stress, involving collaboration between the government and family members to enhance the support system and improve the patient’s quality of life. Second, it is essential to address and improve sleep quality. Enhancing sleep quality can help alleviate stress and reduce the occurrence of depression. Medical staff should focus on alleviating physical discomfort, providing psychological counseling, improving sleep environments, and judiciously administering sleep medications. Finally, there should be a regular assessment of the dynamic psychological state. Healthcare professionals should conduct real-time psychological assessments, and screening, and provide timely interventions for stroke patients, both within and outside the hospital.

### Limitation

While this study has generated valuable results, there are certain limitations. First, the geographical representation of the sample is restricted as participants were sourced from five hospitals in five cities within Henan Province. This limitation hinders the generalizability of the research results to a nationwide context, highlighting the need for future research based on more diverse national data. Second, being a cross-sectional survey, this study cannot establish causal relationships between perceived stress, sleep quality, social support, and depression. This limitation restricts the exploration of dynamic changes in trajectories between these variables. Therefore, future studies should consider adopting a longitudinal research design. Finally, this study identified sleep quality as a partial mediator variable and social support as a moderating variable. This suggests the presence of other variables influencing the relationship between stress and depression. Future research should delve into exploring additional variables affecting this relationship, and consider practical implications for implementation.

## Conclusion

This study identified the moderated mediation of sleep quality and social support between perceived stress and depression, supporting and validating the theoretical framework and hypotheses. The mediating effect of sleep quality and the moderating effect of social support offer a positive perspective for addressing post-stroke depression. Additionally, this study provides a theoretical basis for developing sleep quality intervention programs aimed at improving depression among stroke patients.

## Data availability statement

The original contributions presented in the study are included in the article/supplementary material, further inquiries can be directed to the corresponding authors.

## Ethics statement

The studies involving humans were approved by Ethical approval was obtained from the Ethics Committee of the First Affiliated Hospital of Zhengzhou University (2022-KY-1168-001) prior to this study. The studies were conducted in accordance with the local legislation and institutional requirements. Written informed consent for participation was not required from the participants or the participants’ legal guardians/next of kin in accordance with the national legislation and institutional requirements.

## Author contributions

LG: Funding acquisition, Writing – original draft, Writing – review & editing. MW: Data curation, Writing – review & editing. GN: Writing – review & editing. MZ: Data curation, Writing – review & editing. YX: Data curation, Writing – review & editing. RM: Conceptualization, Writing – review & editing. YG: Methodology, Project administration, Writing – review & editing. YL: Funding acquisition, Supervision, Writing – review & editing.

## Footnotes

^1^ https://www.random-online.com/
